# The Association of Physical Function Measures With Frailty, Falls History, and Metabolic Syndrome in a Population With Complex Obesity

**DOI:** 10.3389/fresc.2021.716392

**Published:** 2021-09-16

**Authors:** Amanda Rhynehart, Colin Dunlevy, Katie Hayes, Jean O'Connell, Donal O'Shea, Emer O'Malley

**Affiliations:** ^1^Weight Management Service, St Columcille's Hospital, Dublin, Ireland; ^2^Obesity Research Group, St Vincent's University Hospital, Dublin, Ireland

**Keywords:** physical function, frailty, falls, falls history, metabolic syndrome, obesity, complex obesity

## Abstract

**Background:** Frailty, falls and metabolic syndrome are known to be associated with poorer physical function. This study builds on available research by further investigating the relationship between physical function measures, including those comprising frailty, with metabolic syndrome (MetS) and falls, in the context of complex obesity.

**Methods:** Participants were recruited from the national Level 3 weight management service in Ireland. A retrospective audit of data gathered at initial assessment was performed. Data included past medical history, blood tests, blood pressure measurement, anthropometrics, falls history, self-reported physical activity levels (PALs) and physical function measures, including hand grip strength (HGS), “timed up and go” (TUG), functional reach (FR), sit to stand (STS) and gait speed. A modified version of the Fried Frailty Index was employed.

**Results:** Of the 713 participants, 65.1% (*n* = 464) were female and 34.9% (*n* = 249) were male with a mean age of 44.2 (±11.7) years and body mass index (BMI) of 50.6 kg/m^2^ (±8.2). Frailty was identified in 3.4% (*n* = 24), falls in 28.8% (*n* = 205) and MetS in 55.1% (*n* = 393). Frailty was associated with older age (53.8 ± 14.3 vs. 43.9 ± 11.5 years), poorer PALs (27.29 ± 46.3 vs. 101.1 ± 147.4 min/wk), reduced grip strength (17.7 ± 4.6 vs. 34.2 ± 11.0 Kg) longer STS (21.7 ± 6.6 vs. 13.7 ± 5.7 s), shorter functional reach (29.7 ± 7.9 vs. 37.9 ± 8.2 cm) and slower gait speed (0.6 ± 0.2 vs. 1.1 ± 0.5 m/s). Those reporting a falls history had a reduced FR (35.8 ± 8.9 vs. 38.3 ± 7.8 cm) and slower STS (15.4 ± 8.0 vs. 13.3 ± 4.7 s). Participants with MetS had lower PALs (83.2 ± 128.2 vs. 119.2 ± 157.6) and gait speed (1.1 ± 0.3 vs. 1.2 ± 0.7 m/s). There was no difference in BMI between fallers and non-fallers (51.34 ± 8.44 vs. 50.33 ± 8.13 Kg/m^2^, *p* = 0.138), nor between those with or without MetS. Significant associations were found between BMI and all physical function measures except the TUAG.

**Conclusion:** The associations between frailty, falls and MetS and their combined impact on physical function in people living with obesity demonstrates the need for appropriate screening. Utilising grip strength and gait speed to identify frailty in those with obesity and metabolic syndrome could help target therapies aimed at improving strength, physical function and ultimately quality of life.

## Introduction

Frailty is a multidimensional concept that is primarily associated with an age-related decline in functional capacity. It has significant implications for an individual's response to stressors (1) including falls. Frailty is linked to poorer health outcomes in older adults (2) and is a risk factor for both single and recurrent falls in those 50 years and over (3). The development of frailty has been associated with both chronic obesity and the development of obesity in late adulthood (4). The Fried Frailty Index (FFI) is the most cited frailty screening tool (5). It is composed of five criteria, including unintentional weight loss, self-reported exhaustion, reduced strength, slow walking speed, and low physical activity.

The Irish Health Service Executive and the World Health Organisation (WHO) state over 1 quarter of community-dwelling adults over 65 years report falling at least once each year (6), while data from the Irish Longitudinal study on Ageing (TILDA) reports falls in a third of Irish adults aged 65 years and over (7). The WHO state that falls are associated with a significant economic burden (8) with between 0.85 and 1.5% of total healthcare expenditure devoted to their management (9). While falls are often considered a problem for older populations, Peeters et al., analysing data from across four population-based cohort studies, highlighted a sharp increase in prevalence of falls in middle-age, suggesting that falls are not just a problem of old age (10). This is also born out in The Irish Longitudinal Study on Ageing (TILDA) report 2019 (7).

Reduced muscle function and abdominal obesity have been associated with metabolic syndrome in older adults (11), which in turn has been associated with falls in this age group (12, 13). There is little data however, on the relationship between muscle function and abdominal obesity in younger adults, or those living with obesity, despite the prevalence of metabolic syndrome in obesity. The association between muscle function, falls has been highlighted primarily as an age-related muscle strength problem (14). However, recent research among people living with obesity has identified an increased risk of falls among this population across age groups (6, 15, 16). As noted in these studies, many questions remain unanswered regarding the impact of obesity on functional capacity in younger adults. The concurrent presence of metabolic syndrome may present an additional mediating factor in this impact.

Obesity is recognised as a complex multifactorial disease affecting a range of body systems. The impact of the chronic low-grade, systemic inflammatory process in obesity on metabolic regulation is well-documented (17), as is reduced physical function in those with obesity (18). Decreased muscle function has been associated with metabolic syndrome across age groups (19) which is a component of falls risk, and frailty.

In recent decades, the need for assessment and timely diagnosis of weight-related metabolic complications in younger adults and adolescents has been recognised (20, 21). However, the same attention is not given to the assessment of frailty and its composite measures in younger people living with obesity. Given the association with metabolic syndrome, frailty may be underdiagnosed in this group. It is possible that frailty is underdiagnosed in younger people with obesity. Identifying the presence of frailty is challenging given its multifactorial nature, however measures of physical function are useful in assessing both frailty and falls risk. Although no one frailty measure has been agreed upon, the Fried Frailty phenotype measure has been extensively used, with gait speed and grip strength combined being used as a proxy measure to determine frailty in primary care (1). Population-specific reference ranges are used to identify natural variance in physical function, for example in age and gender. These provide helpful guidance in relation to identifying at-risk groups. Comparisons between populations with and without obesity, across similar age and gender cohorts, serves to contextualise these measures and inform risk identification in people with obesity.

**Figure 1:**
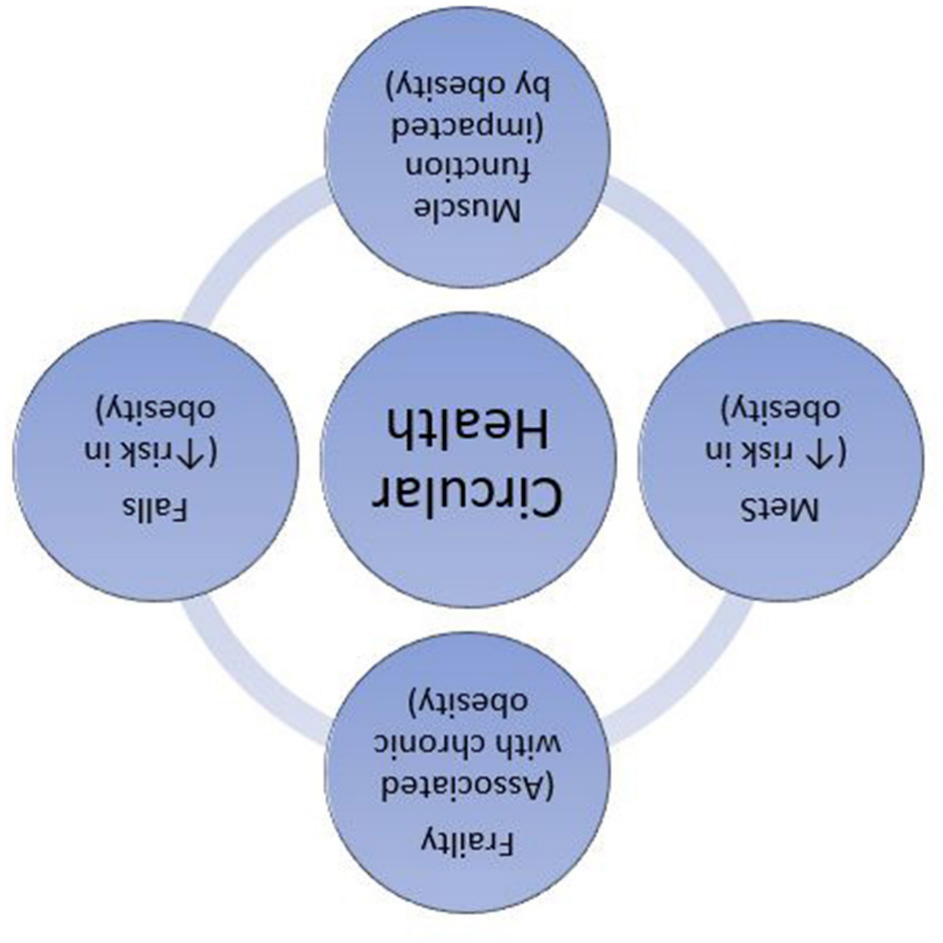
The role of circular health: assessment of frailty, falls, muscle function and Metabolic Syndrome (MetS), in the context of obesity.

The concept of circular health (Figure 1) refers to the key role of an interdisciplinary approach in identifying and treating the multifactorial determinants of ill health. While specialist treatment of complex obesity involves assessment and treatment of both metabolic and physical function parameters (22), there is little guidance on how these assessments should inform each other. Given the interplay between metabolic syndrome, muscle function, falls and frailty, each of these should be considered in the assessment and management of the others. These interactions may be overlooked in this population, highlighting the importance of a circular health perspective when treating complex obesity.

## Aims

This study aims to build on available research into metabolic syndrome, frailty, and falls, by further investigating the relationship between physical function measures, including those comprising frailty, with metabolic syndrome and falls, in the context of complex obesity of Grade 3 and above.

## Methods

A comprehensive multidisciplinary assessment is performed for all patients attending the national Level 3 weight management service (WMS) in Ireland. The WMS delivers a yearlong programme of MDT treatment with medical, nursing, physiotherapy, dietetics and psychology input, including assessment for bariatric surgery. The WMS accepts referrals for adults (aged 18 years and above) with complex obesity from primary and secondary care providers. A retrospective audit of data gathered at initial assessments was performed. Patients attending the WMS record consent for utilisation of personal and medical data for research purposes as approved by the St. Vincent's University Healthcare Group Ethics and Medical Research Committee. Data included blood pressure, triglycerides, HDL cholesterol, fasting blood glucose, past medical history, anthropometric measurements, falls history, self-reported physical activity levels (PALs), physical function measures, including hand grip strength (HGS), “timed up and go” (TUG), functional reach and gait speed. Weight (Kg) and height (m) were measured on a Seca digital scales and stadiometer and body mass index (BMI) in Kg/m^2^ was calculated. Blood pressure was measured using an extra-large automated cuff (Omron HEM-907XL).

For this study, a modified version of the Fried Frailty Index was employed, utilising grip strength in conjunction with gait speed. This dual criteria measure for predicting the Fried Frailty phenotype has been shown to be accurate, with positive predictive value of 87.5% (1). A cut-off value of 0.9 m/s in walking speed and 28 kg in grip strength for men, and a corresponding value of 0.8 m/s and 18 kg for women were used (23). Those below the cut off point for both measures were categorised as frail.

Fallers were categorised as those who had fallen at least once in the preceding year. Physical activity levels were recorded as minutes per week. HGS was measured using a hand-held dynamometer with the upper limb in a neutral position and the elbow flexed to 90 degrees (Jamar®, Patterson Medical, Warrenville, IL, USA) in accordance with the recommendations of the American Society of Hand Therapists Hand Dynamometer (24) and was recorded in kilogrammes. The TUG was recorded as the time taken for an individual to stand up, walk 3 m turn and return to sitting (25). Functional reach, whereby the distance between an initial starting position and maximal forward reach was measured in cm. In standing, participants were asked to raise their arm to 90° of shoulder flexion and to reach forward in the same plane to a maximal forward reach finishing position.

The International Diabetes Federation (IDF) categorises metabolic syndrome as having central obesity and two of the following: raised triglycerides (≥1.7 mmol/L), reduced HDL cholesterol (≤ 1.03 mmol/L for men or ≤ 1.29 mmol/L for women), raised fasting plasma glucose (HbA1c ≥38 mmol/mol) or Type 2 diabetes and hypertension (systolic BP≥130 mm Hg) (26). Those meeting two or more of type the above criteria with a BMI ≥30 kg/m^2^ were categorised as having metabolic syndrome. Waist circumference measurement is often not feasible in this population due to the presence of a panus or apron and is not recommended in those with a BMI over 35 kg/m^2^ (27), and so is not routinely measured at initial WMS assessment. This BMI cut-off was used as it incurs increased cardio-metabolic risk comparable to waist circumference in line with the ICCR consensus statement (28).

Differences in physical function measures were assessed across gender and between fallers and non-fallers and those with and without metabolic syndrome and those with and without frailty using Mann-Whitney tests. Correlations between continuous variables were assessed using Spearman's Rank Correlation Coefficient. To further investigate the relationship between physical function measures, frailty and metabolic syndrome, patients were grouped into three groups: those with both frailty and metabolic syndrome, those with only one of these conditions, and those with neither. Differences in the physical function measures not included in the frailty measure were compared *via* analysis of variance using Kruskal-Wallis tests with *post-hoc* analysis of significant results.

Values of *P* < 0.05 were considered significant. Statistical Package for Social Science (IBM SPSS) (SPSS, Inc., Chicago, Illinois) was used for all statistical analysis. Participants were excluded due to missing data on physical function measures, falls, or metabolic syndrome.

## Results

### Demographics

Data on 713 WMS patients was analysed. The majority were female (female *n* = 464, 65.1% vs. male *n* = 249, 34.9%). Mean age was 44.2 ± 11.7 years, with men slightly older than women (45.3 vs. 43.6 years, *p* = 0.05). Mean BMI was 50.62 ± 8.23 kg/m^2^, median 53.6 kg/m^2^ ranging from 30.6 to 92.0 kg/m^2^. Fallers accounted for 28.8% (*n* = 205), with 71.2% (*n* = 508) non-fallers.

### Comparisons

Normality of distribution was assessed using Shapiro-Wilk test for age, BMI and physical function measures. All were found not to be normally distributed. There was no significant difference in BMI, falls history or frailty between genders.

Frailty was identified in 3.4% (*n* = 24) patients, with no significant difference between genders. Frail patients were significantly older (53.8 ± 14.3 vs. 43.9 ± 11.5 years, *p* = 0.00), had lower PALs (27.29 ± 46.3 vs. 101.1 ± 147.4 min/wk, *p* = 0.003), slower TUG (12.43 ± 4.1 vs. 8.5 ± 14.6 s, *p* = 0.00), and took longer to sit to stand (21.7 ± 6.5 vs. 13.7 ± 5.7 s, *p* = 0.00), and had a shorter functional reach (29.7 ± 7.9 vs. 37.9 ± 8.2 cm, *p* = 0.00). Frailty was associated with falls, with falls significantly more common in those categorised as frail, (50% fallers in frail vs. 28% fallers in “not frail,” Pearson Chi Square *p* = 0.024). Frailty was not associated with presence of metabolic syndrome.

There was no difference in BMI between fallers and non-fallers nor between those with or without metabolic syndrome.

Metabolic syndrome was present in 55.1% (*n* = 393) of patients with no significant difference between genders. There was no difference in the prevalence of metabolic syndrome among fallers (59.5%) compared to non-fallers (53.3%).

Significant correlations were noted between BMI and all physical function measures: self-reported PALs (*p* = 0.000), grip strength (*p* < 0.05), TUAG (*p* = 0.00), functional reach (*p* = 0.001), sit to stand (*p* < 0.01), and gait speed (*p* = 0.000).

Differences in PALs and physical function between genders, fallers and non-fallers, and those with and without metabolic syndrome, and with and without frailty are shown in Table 1. Normality and equality of variance were assessed using Levene's test, and Shapiro-Wilk test. These assumptions were not met, so Kruskall-Wallis test was used to assess physical function measures across the Metabolic Syndrome/Frail groups. All physical function measures [grip strength (*p* = 0.00), TUAG (*p* = 0.00), functional reach (*p* = 0.00), Sit to Stand (*p* = 0.00), and gait speed (*p* = 0.00)] were significantly poorer in those with both frailty and metabolic syndrome and in those with only one condition, compared to those with neither. PALs did not vary significantly across the groups.

**Table 1:** Physical function measures.

	**Total *N* = 1,144** **Mean ± SD**	**Female (*n* = 746)** **Mean ± SD**	**Male** **(*n* = 398)** **Mean ± SD**	***P*-value**	**Frail** **(*n* = 46)**	**Not frail** **(*n* = 1,098)**	***P*-value**	**Faller** **(*n* = 329)** **Mean ± SD**	**Non-faller (*n* = 815)** **Mean ± SD**	***P*-value**	**Metabolic syndrome** **(*n* = 325)**	**No metabolic** **syndrome (*n* = 819)**	***P*-value**
Current PALs (mins/wk)	98.6 ± 145.7	93 ± 141.8	108.7 ± 152.5	0.098	27.29 ± 46.3	101.1 ± 14.4	0.003^*^	93.7 ± 142.7	100.6 ± 147.0	0.497	111.19 ± 157.6	83.2 ± 128.2	0.011017^*^
Mean grip strength (Kg)	33.6 ± 11.2	28.3 ± 7.3	43.5 ± 10.55	0.000^*^	17.7 ± 4.6	34.2 ± 11.0	0.000^*^^∞^	32.9 ± 11.9	33.9 ± 10.9	0.150	34.1 ± 11.8	33.0 ± 10.5	0.558
Mean TUAG (seconds)	8.6 ± 14.4	8.1 ± 2.8	9.6 ± 24.1	0.795	12.4 ± 4.1	8.5 ± 14.6	0.000^*^	8.7 ± 3.8	8.6 ± 16.9	0.030^*^	9.0 ± 19.3	8.2 ± 2.6	0.067
Mean functional reach (cm)	37.6 ± 8.3	36.5 ± 8.0	39.7 ± 8.4	0.000^*^	29.7 ± 7.9	37.9 ± 8.1	0.000^*^	35.8 ± 8.9	38.3 ± 7.8	0.003^*^	38.1 ± 8.8	37.1 ± 7.5	0.074
Mean sit to stand	14.0 ± 5.9	13.6 ± 4.9	14.7 ± 7.2	0.001^*^	21.7 ± 6.6	13.7 ± 5.7	0.000^*^	15.4 ± 8.0	13.3 ± 4.7	0.000^*^	13.7 ± 6.5	14.2 ± 4.9	0.052
Gait speed	1.1 ± 0.6	1.1 ± 0.6	1.1 ± 0.3	0.223	0.6 ± 0.2	1.1 ± 0.5	0.000^^*^^^∞^	1.1 ± 0.9	1.1 ± 0.3	0.015^*^	1.2 ± 0.7	1.1 ± 0.3	0.003^*^

* *Statistically significant difference shown by Mann-Whitney test*.

∞ *Included in frailty measure*.

## Discussion

This study showed associations between physical function and BMI. We found that functional reach and sit to stand measures were poorer in patients with frailty or a history of falls, and that these differences were additive, with significantly poorer functional reach and sit to stand performance in those with both frailty and metabolic syndrome compared to those with only one condition, or neither.

Published data on the Irish longitudinal study on ageing (TILDA) population, a nationally representative sample of community-dwelling adults aged 50 years and above living in the Republic of Ireland, reports prevalence of physical frailty in Ireland increasing with advancing age: 11% in those aged 55+; 15% in 65+; 19% in 70+; 25% in 75+; 35% in 80+ and 46% in 85+ (3). Frailty in this younger population with obesity was markedly less at 3.4%, which is lower than even the 8.6% reported from the Canadian Health Measures Study data estimates of frailty in adults 18–79 years old (29). A prevalence of frailty based on the Fried criteria was 6.5% in a Canadian study of older community dwelling adults (1).

The complete FFI classifies physical frailty status by the presence in an individual of five criteria, namely exhaustion/fatigue, unintended weight loss, slow walking speed, reduced muscle strength and low levels of physical activity. The presence of none, 1–2 and ≥3 of these criteria indicates that an individual is non-frail, pre-frail or frail, respectively (30). Hence, the classification used in this study captures those who were at a minimum pre-frail but may have missed identifying some individuals for whom self-reported exhaustion was a factor.

Despite the low prevalence of frailty, its association with falls identified in this study highlights its relevance in terms of clinical assessment of complex obesity. Frailty negatively influences outcomes after both unintentional stressors such as infection (31) and planned interventions such as surgery (32). Identification of patients with frailty would allow for targeted rehabilitation of patients referred for bariatric surgery.

Wang et al. (33) reported mean grip strength across age and gender cohorts, with wide-ranging results, from 18.7 kg for the non-dominant hand in older women, to 49.7 kg for the dominant hand in younger men. The age group closest to the WMS cohort were those aged 45–49 years, who had a mean grip strength of 42.8 kg ± 10.9 for men and 29.9 kg ± 6.2 for women. These results are similar to the WMS cohort suggesting that determining muscular endurance rather than static measurement alone may be important in identifying at risk groups.

A further meta-analysis on functional reach reported a mean functional reach of 27.2 cm in older adults (34). Previously reported data on younger cohorts provides a closer age range for comparison with the WMS population, with those aged between 41 and 69 years achieving a functional reach of 38 ± 5.6 cm for men and 26.6 ± 9 cm for women (35). The mean functional reach of 37.6 cm in the WMS population compared well with this.

Bohannon in a meta-analysis of normative values for the five times sit to stand measure reported a mean time of 11.4 s in adults 60–69 years, 12.6 s among 70–79 years and 14.8 s in participants 80–89 years (36). Given the mean time for participants in the WMS cohort was 14.0 s, overall performance in this dynamic test was comparable to much older adults.

The TUG is a widely used health outcome measure. The WMS population, with a mean age of 44.2 ± 11.7 years, had an average TUG test of 8.6 (±14.4) s. Normative data published by (37) showed that this value was associated with a population of 60–69 years. By this measure, our population aligns with a group 20+ years older, reflecting the difficulties experienced by this population with everyday mobility.

This is further reflected in the gait speed data. A meta-analysis of gait speed by Bohannon found that among men and women 40–49 years a mean of 1.43 m/s (1.3–1.5) and 1.4 m/s (1.3–1.4) respectively was achieved (38), compared to 1.1 (±0.6) for women and 1.1 (±0.3) for men in the WMS population. The only participants to have slower gait speed than the WMS cohort were men aged 80–99 years, with a gait speed of 0.97 m/sec (0.83–1.10), and women aged 70–77 years, 1.13 m/sec (1.07–1.19) and 80–99 years, 0.94 (0.85–1.03).

Falls are often discussed in the context of ageing, and the consideration of age-related reductions in muscle mass and bone mineral density. However, falls present a risk for people of all ages living with obesity. One in three (28.8%) of WMS patients in this study had a history of falls. The TILDA report on Health and Wellbeing: Active Ageing for Older Adults in Ireland reports a prevalence of 37.3% of any fall reported in the last year (Matthew O'Connell). Comparison with the TILDA data on individual age groupings highlights that falls rate among the WMS population, whose average age is mid-40's, was equivalent to that in over 50's within the general population (39).

Metabolic syndrome among TILDA population was reported between 41.6 and 47.3% depending on the criteria used (40). With substantially higher levels in the WMS population at 55.1%, presence of metabolic syndrome is of concern due to the increased cardiovascular risk and morbidity associated with it (41). Additionally, the association of metabolic syndrome with physical function measures and frailty found in this study, highlights the cross relevance of these measures in this younger population with obesity, and mirrors the association with falls and frailty noted among older adults (13). A pathway of altered body composition, including sarcopenia, leading to reduced physical activity, fitness and functional performance, and eventually resulting in increased risk of metabolic syndrome, reduced Qol and increased risk of falls-related fractures has been outlined in other patient groups, such as those with cancer (42). Sarcopenic obesity with concurrent metabolic syndrome appears to form a similar pathway.

These findings suggest that patients living with complex obesity are performing at a similar level to adults at least a decade their senior. Despite the daily challenges of life experienced by these patients due to this relatively impaired physical functioning, falls risk factors, such as fear of falling, which are routinely considered in older populations (1, 39) are not a routine component of obesity assessment. The addition of monitoring for early risk factors for falls to standard clinical assessment of people living with obesity, could improve prevention and management of these conditions, and support independent living.

The Edmonton Obesity Staging System (EOSS) is a widely used (43) five-stage system of obesity classification that utilises metabolic, physical, and psychological parameters to determine optimal obesity treatment. First introduced in 2009 (44) it is a framework for holistic assessment and encompasses the four main health domains: metabolic, mechanical, mental health, and social milieu (the 4Ms). The EOSS considers comorbidity and functional status in predicting mortality. Using such a framework positions the metabolic and mechanical domains in relation to each other so that the relevance of metabolic syndrome in terms of frailty and falls risk in this population is not overlooked. A framework such as this demonstrates how interventions to assess and support physical function can be positioned across these domains to maximise their impact.

The associations seen in this study between frailty and metabolic syndrome in people with obesity adds to the data available on these common concerns in this high-risk population.

While both metabolic health and physical function are considered in the guidance on specialist treatment of complex obesity (22), their cross-discipline relevance, and combined importance are not specifically noted. Frailty and falls are both multifactorial concerns, with several modifiable risk factors that lend themselves to preventative interventions, including physical function and metabolic syndrome. Screening for frailty and falls through utilising grip strength and gait speed in those with obesity and metabolic syndrome could help target therapies aimed at improving strength and physical function. Combined resistance and aerobic exercise have been shown to be effective and safe in attenuating metabolic syndrome and sarcopenic obesity (45) simultaneously addressing the risk of frailty and falls and the metabolic dysregulation of metabolic syndrome.

## Study Limitations

Due to the retrospective nature of our data analysis, we were required to utilise a modified frailty scale. Although the study cohort had a lower mean age than most research available on frailty, we feel this may have contributed to an under-estimation of frailty prevalence.

## Conclusion

The findings of this study add to the evidence underpinning the importance of a circular health perspective when treating complex obesity, and the benefit of using a holistic assessment approach that incorporates screening of both metabolic and physical function. We recommend that such an approach be employed in the assessment and treatment planning for patients with complex obesity, to facilitate timely and thorough assessment and appropriate treatment of the interconnected concerns of frailty, falls, and metabolic syndrome.

## Data Availability Statement

The raw data supporting the conclusions of this article will be made available by the authors, without undue reservation.

## Ethics Statement

Ethical review and approval was not required for the study on human participants in accordance with the local legislation and institutional requirements. Written informed consent for participation was not required for this study in accordance with the national legislation and the institutional requirements.

## Author Contributions

AR and EO'M: conceived and designed the study and performed the literature review. EO'M and CD: designed the database and conducted the data collection. AR: performed all statistical analysis, provided structure to the initial draught, and contributed to subsequent iterations. EO'M: wrote sections of the manuscript, guided the clinical direction of research findings, and contributed to manuscript editing and formatting. KH and CD: reviewed and edited draughts and providing expertise on physical function. JO'C and DO'S: reviewed and edited draughts providing guidance from a medical perspective. All authors contributed to the article, agree to be accountable for the content, and approved the submitted version.

## Conflict of Interest

The authors declare that the research was conducted in the absence of any commercial or financial relationships that could be construed as a potential conflict of interest.

## Publisher's Note

All claims expressed in this article are solely those of the authors and do not necessarily represent those of their affiliated organizations, or those of the publisher, the editors and the reviewers. Any product that may be evaluated in this article, or claim that may be made by its manufacturer, is not guaranteed or endorsed by the publisher.
